# Role of trust in sustaining provision and uptake of maternal and child healthcare: Evidence from a national programme in Nigeria

**DOI:** 10.1016/j.socscimed.2021.114644

**Published:** 2022-01

**Authors:** Nkoli Ezumah, Ana Manzano, Uchenna Ezenwaka, Uche Obi, Tim Ensor, Enyi Etiaba, Obinna Onwujekwe, Bassey Ebenso, Benjamin Uzochukwu, Reinhard Huss, Tolib Mirzoev

**Affiliations:** a Health Policy Research Group (HPRG) College of Medicine, University of Nigeria Enugu Campus, Nigeria; b Nuffield Centre for International Health and Development, University of Leeds, United Kingdom; c Department of Global Health and Development, London School of Hygiene and Tropical Medicine

**Keywords:** Nigeria, Trust, Maternal and child health services, Low and middle-income countries, Health system's strengthening, Realist evaluation

## Abstract

Despite increasing attention to implementation research in global health, evidence from low- and middle-income countries (LMICs) using realist evaluations, in understanding how complex health programmes work remains limited. This paper contributes to bridging this knowledge gap by reporting how, why and in what circumstances, the implementation and subsequent termination of a maternal and child health programme affected the trust of service users and healthcare providers in Nigeria. Key documents were reviewed, and initial programme theories of how context triggers mechanisms to produce intended and unintended outcomes were developed. These were tested, consolidated and refined through iterative cycles of data collection and analysis. Testing and validation of the trust theory utilized eight in-depth interviews with health workers, four focus group discussions with service users and a household survey of 713 pregnant women and analysed retroductively. The conceptual framework adopted Hurley's perspective on ‘decision to trust’ and Straten et al.‘s framework on public trust and social capital theory. Incentives offered by the programme triggered confidence and satisfaction among service users, contributing to their trust in healthcare providers, increased service uptake, motivated healthcare providers to have a positive attitude to work, and facilitated their trust in the health system. Termination of the programme led to most service users' dissatisfaction, and distrust reflected in the reduction in utilization of MCH services, increased staff workloads leading to their decreased performance although residual trust remained. Understanding the role of trust in a programme's short and long-term outcomes can help policymakers and other key actors in the planning and implementation of sustainable and effective health programmes. We call for more theory-driven approaches such as realist evaluation to advance understanding of the implementation of health programmes in LMICs.

## Highlights

• Implementation research using realist evaluation helps explain health programmes.
• Financial incentives improve trust contributing to increased use of healthcare.
• Training, remuneration and facility upgrades improve health workers' motivation.
• Loss of incentives and remuneration reduces trust, but residual trust can remain.
• Theory-driven evaluations can explain and help improve complex health programmes.

## 1 Introduction

The last decade has witnessed increased attention to the role of implementation research in global health advancing the understanding of how complex health systems intervention work (or not) under real-world conditions (Alonge et al., 2019; Ridde et al., 2020; Theobald et al., 2018). While the body of knowledge reporting empirical and theoretical lessons from theory-driven evaluations and specifically realist evaluations (REs) is increasing (Dalkin et al., 2015; Ebenso et al., 2019, 2020; Emmel et al., 2018; Etiaba et al., 2020; Gilmore, 2019; Manzano, 2016; Mirzoev et al., 2020; Pawson and Manzano-Santaella, 2012; Uzochukwu et al., 2020), it remains limited from the low- and middle-income countries (LMICs). In this paper, we contribute to bridging this knowledge gap by reporting results from a RE which explored the role of trust in the provision, utilization and sustainability of maternal healthcare services in Nigeria.

Trust is important for the effective functioning of the health system and enhances interactions between patients and healthcare providers. Due to trust, patients are willing to utilize healthcare services, share their health needs and also adhere to treatment. (Gilson, 2003; Peters and Youssef, 2016; Russell, 2005). Trust is conceptualized as a reliance on a trustee, which is voluntary and with confidence (Hurley, 2006; Meyer, 2015). Patient-centred definitions of trust such as Lupton’s (1997) point out that in medical encounters, patients' experiences with their healthcare providers are important in promoting trust. Although Peters and Youssef (2016) indicate that trust is based on the assumption that health providers have the expertise to provide appropriate services to patients, key drivers among pregnant women for utilizing services of Traditional Birth Attendants (TBAs) in developing countries include the feeling of confidence, security and satisfaction (Amuta-Onukagha et al., 2017; Ugboaja et al., 2018), which emanate from relational dynamics and familial ties. In Cambodia villagers trust public healthcare providers because they are perceived to be honest, and skilled; while trust in private providers is rooted in the friendly manner, they carry out their treatment, location and accessibility (Ozawa and Walker, 2011).

However, there is limited focus on how trust influences patients’ health-seeking behaviours and service provision in LMICs (Gilson et al., 2005; Peters and Youssef, 2016). Furthermore, trust literature tends to focus on physicians or on “the health system”, neglecting the analysis of trust between healthcare users and members of healthcare teams in maternal and child health (MCH) programmes in LMICs (Sheppard et al., 2004). In developing countries, it is necessary to draw attention to how trust can develop under challenging circumstances such as limited infrastructure and lack of health workers (Peters and Youssef, 2016). The continuous nature of the prenatal to postnatal care relationship provides an ideal opportunity to examine how trust works regarding the provision and utilization of MCH services in Nigeria.

This paper reports our analysis of how trust works for healthcare users and providers and how it impacts their behaviours and practices in the utilization and provision of MCH services in Nigeria. This is done by using Hurley’s (2006) perspective on ‘decision to trust’ and Straten et al. (2002) framework of factors influencing public trust, and social capital theory (Agampodi et al., 2015, De Silva et al., 2007) to understand data gathered in our evaluation of a national Subsidy Reinvestment and Empowerment Programme (SURE-P/MCH) in Nigeria, which focused on improving access to MCH. This paper aims to explore how, why and under what circumstances trust influenced the uptake of MCH during the SURE-P/MCH programme and may influence continued MCH service uptake after the suspension of the programme. This enables us to showcase a wider utility of RE in explaining and improving the understanding of whether and how complex health systems interventions work within their contexts under real-world conditions (Alonge et al., 2019).

The paper is structured as follows: after providing an overview of conceptualisations of trust and the organization of the health system in Nigeria, we present the methods, report results, discuss our findings, reflect on a wider utility of RE for advancing the understanding of the implementation of complex health programmes in LMICs, provide conclusions and implications for policy and practice.

### 1.1 Types of trust

Three main types of trust (interpersonal, public and workplace) are identified in the literature. Interpersonal trust refers to the trust an individual has in another person for example between a patient and healthcare provider, while, public trust is that placed by people in an institution/system (Gilson, 2003, 2006; Russell, 2005) Workplace trust refers to trust in colleagues, supervisors and employing organizations (Gilson et al., 2005; Okello and Gilson, 2015). To understand the role of trust in influencing the use of MCH services in LMICs it is important to focus on the interconnection between interpersonal and public trust (Meyer et al., 2008) as well as the workplace. Trust, which arises at the interpersonal level is important to sustain public trust that occurs at the institutional level (Gilson, 2003). Workplace trust on the other hand is important for staff to provide round the clock services because it positively impacts their motivation (Okello and Gilson, 2015).

### 1.2 Nigerian health system and MCH

Nigeria is the largest country in Sub-Saharan Africa (SSA) with a complex three-tier decentralized health system, (primary, secondary and tertiary). Private hospitals exist and are owned by individuals and organizations. Before the introduction of western medicine in Nigeria, pregnant women were assisted by their mothers or mothers-in-law in delivery, while traditional birth attendants (TBAs) provided help if they had complications. TBAs handle about one-third of deliveries that occur outside health facilities in Nigeria and are conversant with the cultural practices associated with delivery. Hence pregnant women are confident, feel secure and satisfied using their services (Amuta-Onukagha et al., 2017; Fagbamigbe and Idemudia, 2015; Ugboaja et al., 2018). This substantial role of TBAs indicates a lack of trust in MCH services provided in public health facilities (Ugboaja et al., 2018).

Improving MCH care in Nigeria is a national priority. Access to skilled birth providers continues to elude many pregnant women with wide disparities according to state, rural/urban location, education and wealth status (NDHS, 2018). In response to poor MCH indices in Nigeria, the Federal Government implemented the SURE-P/MCH programme between 2012 and 2015. The supply-side component aimed at expanding access to quality maternal health services and improving MCH outcomes through recruitment, training and deployment of 2000 skilled midwives and 11,000 community health extension workers (CHEWs), supplies and medicines, infrastructure development, and activation of ward development committees (WDCs) in rural communities. The demand side aimed to increase the utilization of health services during pregnancy and at birth by providing conditional cash transfers (CCTs) to pregnant women at public primary healthcare (PHC) facilities.

## 2 Methods

### 2.1 Study design

This paper report results from a component of a broader mixed-methods theory-driven study (Mirzoev et al., 2016), that adopted a RE approach to examine the relationships between contexts, mechanisms and outcomes of community health workers (CHW) programme in Anambra State, Nigeria. It complements our previous reports of focused theoretical lessons (Ebenso et al., 2019; Mirzoev et al., 2020) and empirical results on advocacy for maternal and child health in Nigeria (Okeke et al., 2021; Uzochukwu et al., 2020), security in the provision and utilization of maternal healthcare (Etiaba et al., 2020) and health worker motivation (Ebenso et al., 2020).

RE is a theory-driven approach that guides the implementation of complex interventions through iterative theory development, testing and refinement (Pawson and Tilley, 1997; Robert et al., 2012; Wilson and McCormack, 2006; Wong et al., 2017). Programme theories developed within realist studies explore which contexts trigger which mechanisms that produce intended or unintended outcomes in different contexts. This enables a clear understanding of the ‘whys’ and the ‘hows’ of programme outcomes within a particular context that is well suited to evaluating programmes implemented at diverse levels of the health system investigations in low-resource settings (Marchal et al., 2012). In RE, data extraction proceeds from baseline enquiries and development of programme theory to testing/refinement and consolidation of the programme theory, using empirical data (Dalkin et al., 2015).

### 2.2 Data collection and analysis

The study was conducted in three phases, corresponding to the building of initial programme theories (IPTs), testing/validation and consolidation/refinement (Manzano, 2016; Pawson and Manzano-Santaella, 2012; Pawson and Tilley, 1997) (see Table 1 in supplementary file).

In phase 1, we reviewed key SURE-P/MCH programme documents and relevant MCH Federal and state policies, between June and September 2015, to understand the programme architecture and design (Ebenso et al., 2019). Initial qualitative interviews were held (May–November 2016) with purposefully identified 96 stakeholders comprising in-depth interviews (IDIs) with 10 policymakers, 11 programme officers, 16 health workers/PHC staff, and 15 facility managers at federal and state levels. Focus Group discussions (FGDs) were held with 8 Village Health Workers (VHWs), 12 WDCs, 12 service users (pregnant women) and 12 family members of service users. Different numbers of interviewees reflect the three phases of our research and different engagements with the Trust theory. The selection of all these different respondents was done to explore their views and experiences of the SURE-P/MCH programme. These data facilitated the development of 8 programme theories for the main study (Mirzoev et al., 2016).

In phase 2, which entailed data testing and validation of trust theories, qualitative and quantitative methods were utilized. Data were collected in 12 PHCs and three general hospitals purposively selected to reflect the implementation of the SURE-P/MCH programme in Anambra state, Nigeria. These facilities were clustered into three, each cluster comprised one general hospital and four PHCs. The focus on the clusters reflects the setup of the SURE-P/MCH programme. Two of the clusters benefitted from the SURE-P/MCH intervention, while the third cluster was used as a control. This was relevant to enable us to determine if there were any differences in MCH service utilization in the clusters that benefited from the intervention compared to the control cluster. For the qualitative methods, 8 IDIs with health workers and 4 FGDs with service users were conducted (August–September 2018).

The IDIs and FGDs were guided by a semi-structured question guide designed around the different versions of initial programme theories and included questions for testing and validating the different components of the programme theories for the main study. The FGD interviews were conducted in the Igbo language, while the IDIs were conducted in both Igbo and English languages depending on respondents' preference. All IDIs and FGDs were conducted face-to-face and were audio-recorded with respondents’ consent, transcribed and translated into English as necessary. Female researchers (NE, (Sociologist), UE, (Health Economist), UO and EE (Medical doctors) trained in realist interviewing undertook the data collection while NE, UE, UO, TE (male Health economist), EE and AM (female Sociologist) were involved in data analysis.

Qualitative data collection was complemented by data from a quantitative household survey. The survey was based on a community listing of all households in the project cluster areas that had a birth in the last 6 years; covering a period before, during and after the SURE-P/MCH programme. A stratified random sample of 713 women was selected for quantitative interviews across the three project areas. A questionnaire was administered, which collected information on maternal health-seeking behaviour to the care given and socioeconomic information on the household between May and June 2018.

In phase 3, we refined and modelled the complex relations between the actors, contexts, intervention processes and mechanisms, and its outcomes (December 2018). Using the Context-Mechanism-Outcome (C-M-O) configuration, we examined the emerging data on trust to make inferences about the relationships between contexts, mechanisms and outcomes (Fig. 1). We examined the quantitative data critically to explore the effect of the intervention on various sub-groups of women and to identify sub-period variation in outcome relative to the period before, during and after the withdrawal of the programme. Patterns across data sets were identified by accumulation (the same factor was present within a set and across sets) and causal relationships were established with further support of the theoretical literature and our qualitative data set. This enabled us to refine and consolidate our programme theory on trust which states as follow:

In the context of improved staff attitude, upgraded health facilities and functioning WDCs achieved during the implementation of the SURE-P/MCH programme, pregnant women who receive sustained financial and non-financial incentives to use MCH services (Context), are likely to develop and maintain a sense of improved trust (including confidence and satisfaction) with health facilities and staff (Mechanism), ultimately leading to the improved likelihood of repeated and regular utilization of MCH services from these health facilities (Outcome).

Fig. 1: CMO template visualizing the causal linkages among contexts (Cs), Mechanisms (Ms) and Outcomes (Os) (See Fig. 1 in supplementary file).

### 2.3 Theoretical framework

To explain how trust works, we drew upon Hurley’s (2006) perspective on the ‘decision to trust’ and Straten et al. (2002) framework of factors that influence public trust in healthcare systems. We also utilized social capital theory (Agampodi et al., 2015; Bourdieu, 1986; De Silva et al., 2007). in our interpretation of the sustainability of trust during the SURE-P/MCH Programme and the existence of residual trust by service users after the withdrawal of the programme. Elements of Hurley’s (2006) perspective relevant to explaining trust in LMIC context include security; the number of similarities between the trustee and truster; if the trustee shows benevolent concerns, trustee's capability to do their work.

Straten et al. (2002) provide a useful framework in the explanation of factors influencing public trust at micro, meso and macro levels. They specify that at the micro-level, people are more concerned about the behaviour of the healthcare providers, whether they will listen to them and handle their problems appropriately. At the meso level, peoples' concern is whether the health providers are cooperating among themselves; at the macro level, people are worried about impacts of interventions accompanying the development process in the society on their access to, as well as the quality of healthcare. This framework is relevant in the analysis of trust in LMICs. Peters and Youssef (2016) indicate that at the micro-level, the interaction between the doctor and patient can become more effective based on trust and consequently will enhance the patient's satisfaction and compliance with treatment while at the macro level, the importance of trust is expressed in the impact it makes in society by influencing efforts being made to meet societal expectations. Studies on trust in Nigeria (Amuta-Onukagha et al., 2017, Fagbamigbe and Idemudia, 2015; Ugboaja et al., 2018), reinforce the relevance of articulating factors at the micro, meso, and macro levels in the analysis of trust in health systems.

Social capital is conceptualized regarding entitlements to resources including information, financial benefits, favours and services individuals get through membership to a community and participation in networks. It also implies an expectation of reciprocity among members of the networks (Bourdieu, 1986; De Silva et al., 2007). Hence social capital is perceived as ‘tangible’ and ‘intangible’ resources that members of a group have access to on account of their membership to the group (De Silva et al., 2007). Three types of ties namely ‘bonding’, ‘bridging’, and ‘linking’ social capital are identified. Bonding social capital refers to relationships of trust and cooperation, with strong ties among people who have shared identity such as ethnicity, social class, age and place of residence. The bonding ties serve as means through which individuals seek help and support from members of the network (Erickson, 2011). Bridging social capital derives from respect and mutual relationships in networks that are not homogeneous (Erickson, 2011; Putnam, 2000), while linking social capital, on the other hand, refers to “vertical” ties existing among people who belong to different levels of power in the society (Erickson, 2011).

Three dimensions of social capital are structural, cognitive and relational social capital. Structural capital refers to the existence of social networks through which people have access to resources, people, roles, rules and procedures (Bourdieu, 1986). Cognitive social capital refers to people's perceptions and interpretations of the shared relationships in the networks. The relational social capital is concerned with the nature of personal relationships existing among people through interactions in the social system as well as feelings of trust in the network (Claridge, 2018, Harpham and Grant, 2002).

Although the social capital theory has its origin in developed countries, it has been successfully used in the analysis of health behaviour and outcomes in some LMICs including Nigeria (Agampodi et al., 2017; Lau et al., 2020, Ozawa and Walker, 2011; Semali et al., 2015; Ware et al., 2009). The social capital theory demonstrates that health outcomes are dependent on income inequality levels with a greater impact on communities where inequality is higher and safety nets lower (Rodgers et al., 2019; Vincens et al., 2018) in most LMICs. Therefore, social capital plays a vital role in increasing the levels of trust in the analysis of health behaviour and outcomes in some LMICs and is relevant to our study in Nigeria.

## 3 Results

Two broad themes were identified following analysis of qualitative and quantitative datasets: trust during the SURE-P/MCH programme; and distrust following the withdrawal of government support and funding to the SURE-P/MCH programme. The first theme of trust during the SURE-P/MCH programme is further subdivided into trust by service users, and trust by health workers; whereas the second theme of withdrawal of support and distrust due to withdrawal of services is subdivided into distrust by service users and distrust among health workers. The themes and corresponding sub-themes are presented next.

### 3.1 Trust during the SURE- P MCH programme

#### 3.1.1 Trust by service users

The household survey which investigated how women's trust in the health system changed with the introduction of SURE-P/MCH shows that 25% of those living in SURE-P/MCH areas and 55% of those living in CCT areas said that their trust in the health system increased as a result of the resources provided through the SURE-P/MCH programme (Fig. 2, Supplementary file).

To interpret these quantitative findings, our qualitative data showed that the availability of skilled birth attendants and drugs seemed to enhance trust and service utilization. These combined with a positive attitude of the staff towards the patients, triggered service users' satisfaction and confidence in health workers; and contributed to interpersonal trust between the staff and service users, while also increasing public trust in the health system, ultimately leading to enhanced utilization of the facilities, during the programme: “*I would trust the health centre and come because the nurses were good and would give you good drugs. When they have treated your child and seen that they are effective, you would advise other people to come here*” *(*FGD - Service users, Trader, aged 27). This illustrates aspects of Hurley’s (2006) factors such as “confidence and satisfaction” which enable people to decide to trust as well as aspects of Straten et al. (2002) dimensions of trust such as “trust in the patient-focus of healthcare providers”; “trust in the expertise of healthcare providers”; and “trust in the quality of care”. This finding also depicts relational social capital, highlighting the interpersonal trust existing between the service users and the healthcare providers due to the positive attitude of the latter. Moreover, the existence of this relational social capital emanated from the public trust the service users had in the health system because of the tangible resources they were accessing such as good drugs provided free by SURE-P/MCH.

In the Nigerian context, a lack of confidence in healthcare systems has been linked to the non-availability of high-quality drugs and this has a significant role in MCH (Amadi and Tsui, 2018). Our data demonstrate that the provision of subsided services including some free drugs triggered a sense of removal of the financial barrier, which is a major impediment to accessing healthcare, thus contributing to increased public trust of service users in the health system, increased access to care and enhanced utilization of MCH services.

*… We received drugs here during the SURE P MCH programme free …, they didn't collect money from us because the drugs were free then* (FGD - Service user, farmer, aged 35).

Counterfeit medications purchased by users and/or provided by healthcare staff by unregulated drug outlets (Nyaaba et al., 2021) is also a known characteristic of the Nigerian healthcare system and this fear of counterfeits seemed to get reduced and impacted trust:

*… I come here based on my trust that they don't give fake drugs and so I have 100% trust in them …* and for the safety of the child: *… I come to give birth safely …* (FGD - Service user, poultry farmer, aged 27).

Service users received some incentives during ANC registration, antenatal care (ANC) and postnatal care (PNC). CCT payments ranging from N1000 to N5000 (about USD 30) were given to pregnant women who met the required programme conditions. Also, “Mama Kits” containing toiletries were given to pregnant women during or after delivery. These incentives facilitated access to services that built the trust of women in the system and contributed towards increased and repeated utilization of MCH services. “*The incentives were good, it raised people's spirit …, so the trust of people increased as well as the number of people who came to the health centre”* (FGD - Service users, trader, aged 27).

The programme established and recruited members of WDCs and trained them on sensitizing their target beneficiaries about the programme. It also recruited, trained and incentivized VHWs on creating awareness of the programme and, getting pregnant women and children under the age of five to enroll and utilize the services. This led to bonding social capital between the women in the communities and VHWs, which triggered pregnant women's utilization of the PHC facilities. The provision of incentives to pregnant women who accessed MCH services in the PHC facilities enhanced their satisfaction and confidence in the health system.

… *from the period of SURE- P … they had people who went from house to house who informed us about the health centre … They would encourage women to go to the health centre and see for themselves, they came around to help out at the clinic …* (FGD - Service users, farmer and trader, aged 32).

*Why I am confident coming here is that sometime last year, my child was sick and then a woman encouraged us to come here. We did and they took good care of him* (FGD - Service users, tailor, trader, farmer aged 37).

### 3.2 Trust by health workers

The programme recruited, and trained midwives and CHEWs to provide MCH services. These health providers were deployed to all the implementing health facilities and the national government was responsible for their remuneration. The programme staff were reported to be paid their monthly salaries regularly, which is not always the case in Nigerian public health facilities. Consequently, staff were highly motivated and this triggered workplace trust, which made them available, leading to improved quality round-the-clock services, while also ensuring the availability of skilled birth attendants. Regular staff payment by SURE-P/MCH was a trigger for motivation considering that many trained nurses were previously unemployed as reported elsewhere (Ebenso et al., 2020). Other factors such as the provision of incentives for service users and free drugs which enhanced utilization of MCH services, equipment, accommodation, increased staff strength and training as well as intrinsic joy in having patients to attend to, contribute to the motivation of health workers. This is exemplified by the quote from a health worker in charge of a facility:

*When you talk about staffing, Sure-P made the standard of the workers to be superb. You'd see all mix of staff; midwives, CHEWs … and everybody will be in their uniform while working … Apart from that … Sure-P brought water, they sank boreholes that use solar and not electricity everywhere … Sure-P also brought drugs. The poor people … can now come to the facility* (IDI, Health worker/ Officer in charge, PHC).

### 3.3 Withdrawal of support and distrust due to withdrawal of services

#### 3.3.1 Distrust among health workers

Withdrawal of the programme brought about laying off of all SURE-P/MCH staff and discontinuation of training and supervision. At the individual level, the remaining staff experienced increased workloads, inability to meet financial obligations and lack of motivation, which triggered health workers’, frustration and unwelcome attitude toward the patients. These ultimately contributed to poor service delivery and reduction in utilization of MCH services across the implementing facilities.

A health worker narrated that,

*… when staff are demoralized, they won't come to work when they are supposed to come. …, and you will now discover that people may come to access healthcare services without seeing anybody that will attend to them* (IDI, health worker/Community Health Officer, PHC).

The discouragement experienced by health workers affected their attitude to service users. “*… sometimes you would meet hostile nurses who use abusive words on pregnant women”* (FGD - Service users, Teacher, aged 34).

#### 3.3.2 Distrust by service users

At the organizational level, the withdrawal of the programme incentives triggered a reduction in the supply of medicines and other commodities and the re-introduction of user fees. Qualitative data show that withdrawal of incentives triggered dissatisfaction, decreased sense of financial security leading to distrust in the health system and subsequent reduction in utilization of MCH services. *“As of then (during SURE-P programme), the clinic was filled with people due to the incentives but now, everyone has run away”* (FGD - Service users, Farmer aged 29).

Some users began to visit other service providers, including TBAs while the few that utilized the facilities resorted to purchasing medicines outside the health facilities. This trend was highlighted by a health worker:

*Those things that we need (e.g., drugs and Mama Kits …. which SURE-P used to supply) made people come to the health facility, but now that there is no SURE-P, they are not so motivated, …Now when they come and see that the price is high, it makes them either go to the market or do self-treatment …* (IDI, health worker/Community Health Officer, PHC).

The fact that the withdrawal of incentives and consumables resulted in a reduction in utilization of PHC facilities by pregnant women led to the loss of public trust in the health system is consistent with some studies from Nigeria that identified as barriers to utilization of skilled care in formal healthcare facilities the cost of seeking healthcare and lack of trust in the health system among others (Amuta-Onukagha et al., 2017, Fagbamigbe and Idemudia, 2015; Ugboaja et al., 2018).

Quantitative data show that when women were asked to indicate their level of trust in the health system two years after SURE-P//MCH was ended, of those women that said their trust had increased as a result of SURE-P/MCH, around 33% said their trust in the system had eroded while the remaining 66% said their trust remained the same or had even increased (Fig. 3, Supplementary file).

#### 3.3.3 Residual trust by service users

However, despite the withdrawal of the programme and its incentives, both quantitative and qualitative data show that some service users still have confidence in the health system and maintained unwavering trust in health providers due to their satisfaction and the level of interpersonal trust previously built on the health providers. *“ …. Some of them (service users) that came in during SURE-P/MCH continued may* *be because they were pleased with what they saw, then convincing others to come in while the SURE-P had already closed, but some still trust them and come”* (IDI, health worker/Nurse-Midwife, PHC).

*For me, it wasn't because of the incentives but the fact that if I am sick, they would treat me well but there are those that lost trust because the incentives had stopped, so they were going somewhere else* (FGD, service user, fruit trader 29 years).

This buttresses Straten et al. (2002) perspective that at the micro-level people are more concerned about the positive behaviour of the healthcare providers and whether they will handle their healthcare needs appropriately. FGD responses also highlight the relevance of social capital theory in the explanation of the existence of residual trust experienced in our study. The residual trust depicted in both qualitative and quantitative data conform to the social capital theory about how ‘bonding’ and linking social capital led to trust. The bonding ties serve as a means of enabling service users to seek help from the healthcare providers to pay for treatment in instalments, which is not usually applicable in other health facilities. Bonding and linking social capital enhance interpersonal trust between healthcare providers and service users and consequently results in the residual trust identified in our study.

## 4 Discussion

In this paper, we provided an example of how REs can help explain and improve our understanding of whether and how complex health programmes are implemented in the real world of LMICs, including the consequences when programmes are withdrawn. We have done so by exploring how, why and in what circumstances, the implementation and subsequent termination of the SURE-P/MCH programme affected the trust of service users and health service providers in Nigeria. Specific issues in the findings related to sustainability of trust in the health system due to services and incentives provided during SURE-P/MCH and lack of trust, due to the withdrawal of services and incentives, are discussed.

### 4.1 Sustainability of trust in the health system

SURE-P/MCH programme ushered in staff training and re-training; provision of monetary and non-monetary incentives; subsidization of MCH services, including drugs; facility upgrade; and improved staff remuneration. Health providers are motivated when there is institutional support through the provision of resources such as equipment, upgrading of health facilities and in-service training, and regular remuneration as occurred during the programme. This facilitated the development of health providers' public trust in the health system and workplace trust. This development led to the provision of quality care by well-motivated staff and contributed to increased utilization of services and building of interpersonal trust in health workers and public trust in the health system by service users. This finding corroborates the reports of an integral relationship between positive staff attitude and patient-provider interpersonal trust, service users’ satisfaction and utilization of health facility services (Gilson et al., 2005; Gopichandran and Chetlapalli, 2013; Nwabueze et al., 2011).

Provision of financial and non-financial incentives resulted in increased utilization of MCH services amongst users due to the development of trusting behaviour in the health system. This reflects literature on the impact of incentives on the utilization of MCH services. Ekezie et al. (2017) report that incentives enable women to take care of barriers arising due to user-fees that are burdensome and motivate them to seek healthcare and in turn improve their standard of living. The use of WDCs and incentivization of the VHWs enabled programme awareness creation, mobilization and support of women in the communities to utilize MCH services. This led to trust in the health system as well as satisfaction among the service users and corroborates Thomson et al. (2012) report that incentives promote interpersonal relationships between providers and consumers.

### 4.2 Withdrawal of support and distrust due to withdrawal of services

The termination of the SURE-P/MCH programme was accompanied by a withdrawal of programme incentives and a re-introduction of user fees. Withdrawal of access to free drugs by the SURE-P/MCH programme poses a serious concern for service users as it predisposes them to the risk of using TBAs for MCH services (Okonofua et al., 2018) and counterfeit drugs. Access to genuine drugs in Nigeria is a major health challenge due to the proliferation of counterfeit drugs (Akinyandenu, 2013). Withdrawal of these programme incentives compromised the quality of care due to the deterioration of MCH services. This triggered dissatisfaction, consequently leading to distrust in the health system and subsequent reduction in utilization of MCH services. Graham et al. (2015) report that good quality services are associated with the possibility of retaining existing clients and attracting new ones. Our study shows that the withdrawal of drugs and other support services led to the loss of public trust in the health system which, resulted in reduced utilization of MCH services, as service users could no longer rely on the health providers and the health system with confidence. Our data reflect other studies (Ugboaja et al., 2018) that distrust in the health system is a barrier to service utilization and results in poor health outcomes. Baba-Ari et al. (2018) demonstrate that although CCTs influenced the uptake of MCH interventions by beneficiaries in North Central Nigeria, that lack of trust was one of the factors that constrained uptake of the programme by non-beneficiaries.

Factors associated with the withdrawal of the SURE-P/MCH programme namely reduced staff strength; increased workloads; lack of training and supervision; and reduced income made service providers lose trust in the health system. This manifested as poor staff attitude towards patients. Okello and Gilson (2015) report that low remuneration and inadequacy of resources trigger workplace distrust and demotivates health workers in LMICs.

However, we also observed that the majority of household survey respondents who had indicated that the programme increased their trust also said that withdrawal made no difference. This suggests that for many households, the trust built up during the programme persisted well beyond the end of formal financial support. Similarly, some of the service users had unwavering trust towards the service providers despite the withdrawal of the SURE-P/MCH programme and its accompanying incentives. The existence of this residual trust could be attributed to unchanged staff positive attitude in those facilities. This highlights relational social capital, depicting the interpersonal trust existing between the service users and the healthcare providers due to the health workers' positive attitude to their work and care of their patients. It also corroborates the perspective of Straten et al. (2002) that at the micro-level people are more concerned about the behaviour of the healthcare providers, focusing on whether they will handle their needs appropriately. Furthermore, the residual trust could be attributed to the existence of ‘bonding’ and linking social capital between the healthcare providers and the service users. Most of the healthcare providers reside in the communities studied and share similar cultural and social identities with the service users. Therefore, the bonding and linking social capital enable service users to seek help and support from the healthcare providers as demonstrated in requests for instalment payment for treatment by some service users. The existence of bonding and linking social capital is also attributed to trust that was established as a result of repeated interaction This in turn enhanced interpersonal trust between healthcare providers and service users and consequently triggered the residual trust identified after the withdrawal of the programme.

### 4.3 Utility of realist evaluation in implementation research

In this paper, we used a realist evaluation approach to understand how trust affected the utilization and provision of maternal healthcare services in Nigeria. This allowed us to unpack the black box and gain a deeper understanding of how the programme worked in real-world conditions. The theory-driven mixed-method approach utilized quantitative and qualitative evidence to evaluate what works, in what circumstances, for whom and for how long. As Ridde et al. (2020) stated, complex evaluation is about analysing real-world implementation by “providing critical evidence about the implementation process and its outcomes about, and not in isolation from, other elements of the context that may influence the intervention”. This paper demonstrates how trust in healthcare systems is a dynamic force, which is intertwined between users and healthcare staff trust in LMICs including Nigeria. Our paper contributes to an argument in support of a wider utility of theory-driven evaluation approaches such as REs in explaining and improving the implementation of complex health programmes and interventions from LMICs, considering multiple contextual influences leading to expected and unexpected outcomes. Although the body of empirical and theoretical work on REs is increasing, we call for more realist research, particularly from LMIC settings.

### 4.4 Strengths and limitations

• RE methodology has been useful in evaluating the SURE-P/MCH programme. This facilitated the development of initial programme theories and enabled us to explore how and why and in what circumstances the context of SURE-P/MCH programme implementation triggered mechanisms that generated anticipated and unanticipated outcomes within the system.

### 4.5 Limitations

• The theoretical framework is based on theories developed in Western rich and industrialised countries (Gilmore, 2019) and potentially may miss apprehending the contextual systemic factors embedded in LMICs and Nigeria. Social capital theory, however, is based on the notion of actor-based capital to understand mechanisms of inequality which are fundamental in understanding LMICs' health systems.
• Questions in the qualitative interview guide did not directly address ‘trust’ but ‘factors’ that determine trusts such as confidence and satisfaction. That might have affected the comprehensiveness of the responses. Since the interviews were conducted after the withdrawal of the SURE-P/MCH programme, the time lag could have affected the recall of experiences of the interviewees. Moreover, trust and confidence are interrelated in the sense that both have an underlining notion of expectation. While we regard satisfaction as an outcome of trust conceptually, different respondents saw trust and satisfaction as being interrelated.

### 4.6 Implications for policy and practice

Based on the findings presented in this paper, we recommend that government and policymakers need build-in sustainable structures to support the real-world implementation of policy designs, and to mitigate sudden programme withdrawal and its subsequent effects on service users and service providers. In LMICs for example this may include, fee exemption, provision of free drugs in PHCs to enable the poor and vulnerable to have access to quality drugs rather than being exposed to the risk of cheap counterfeit drugs; provision of regular and adequate remuneration to health workers to motivate them to have a positive attitude to their work and service users. This would enhance interpersonal trust between the health workers and service users as well as public trust in the health system by health providers and service users, and workplace trust for health providers.

## 5 Conclusion

Contextualisation of global health interventions in implementation research and the detail of context must be made explicit (Theobald et al., 2018) to ensure generalisable knowledge while operating under context-specific problem-solving. REs have an important place in helping to explain and improve our understanding of whether and how complex programmes or interventions are implemented and work in the real world of LMICs, and we call for more realist research from LMICs. The SURE-P/MCH programme was beneficial due to subsidized services and drugs, financial and non-financial incentives. This encouraged service users to trust the healthcare providers and the health system and increased service utilization and removed financial barriers to accessing MCH services. However, withdrawal of the programme led to distrust in the healthcare providers and the health system and reduced utilization of MCH services. Our findings will be useful to government and policymakers in designing policies and practices for implementing health programmes that will enhance interpersonal trust and public trust in the health system by health providers and service users, and workplace trust for health providers. This in turn will trigger the provision of quality healthcare services and increased utilization of health facility services in Nigeria.

## Authors contribution

The study was conceptualized by TM, AM, OO, BU and BE. Data collection was undertaken by NE, UE, UO and EE while data analysis was carried out by NE, UE, TE, EE and AM. The first draft of the manuscript was prepared by NE, UE and UO. All the co-authors participated in revising and editing the manuscript and all co-authors also gave approval of the final version of the manuscript.
