# Supplementary material for: Role of trust in sustaining provision and uptake of maternal and child healthcare: Evidence from a national programme in Nigeria
Source: Soc Sci Med. 2022 Jan;293:114644. doi: 10.1016/j.socscimed.2021.114644 (PMC8819156; doi:10.1016/j.socscimed.2021.114644)
Supplement: Multimedia component 1 [file mmc1.docx]

**Supplementary file of Figures**


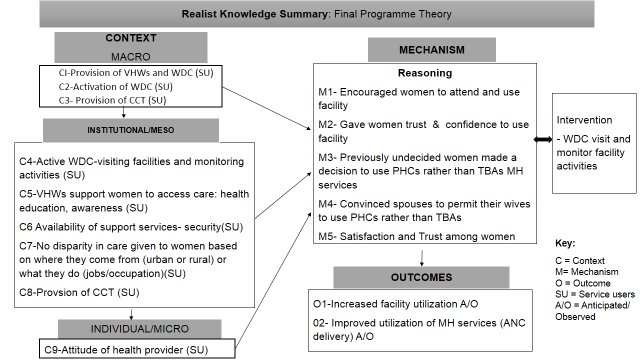


**Figure1: CMO template visualizing the causal linkages among contexts (Cs), Mechanisms (Ms) and Outcomes (Os).**

**(No colour for figure1 in print)**

Figure 2: Change in trust once SURE-P was introduced (% of women)


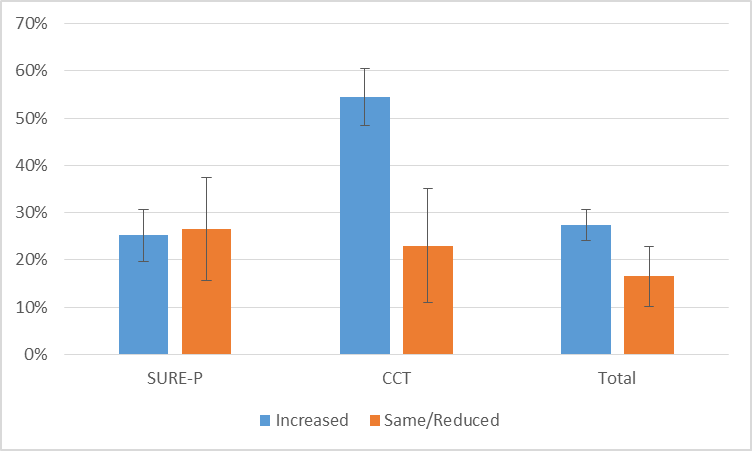


Note: Error bars represent 95% confidence intervals

***(Retain colour for print in figure 2)***

**Figure 3: Change in trust when SURE-P was withdrawn**


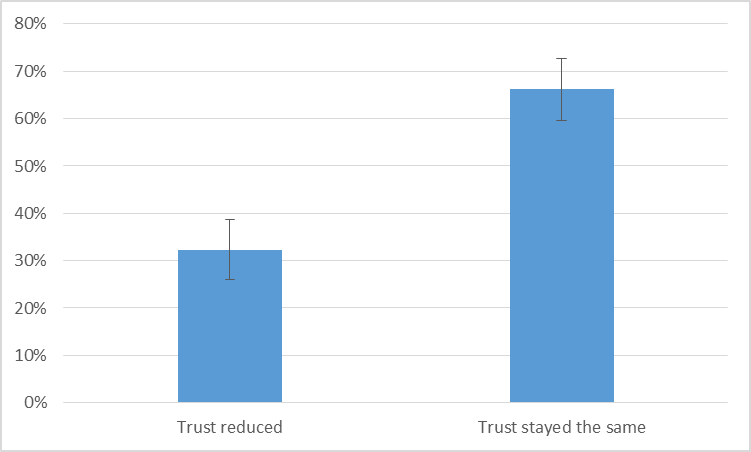


**Note: Of women indicating that their trust in the system increased when SURE-P was introduced**

***(No colour for figure 3 in print)***
