# Supplementary material for: Role of trust in sustaining provision and uptake of maternal and child healthcare: Evidence from a national programme in Nigeria
Source: Soc Sci Med. 2022 Jan;293:114644. doi: 10.1016/j.socscimed.2021.114644 (PMC8819156; doi:10.1016/j.socscimed.2021.114644)
Supplement: Multimedia component 2 [file mmc2.docx]

**Supplementary File of Tables**

**Table 1: Showing the three phases of methods of data collection**

| Phases | Features | Methods of data collection |
| --- | --- | --- |
| Phase 1 | The building of initial programme theories (IPTs) and programme theories | (i)Review of SURE-P/MCH programme implementation manual, relevant federal and state-level policies) (June – September 2015)  (ii)IDIs with stakeholders: 10 policymakers, 11 programme officers, 16 health workers/PHC staff, and 15 facility managers at Federal and state levels (12 PHCs and 3 General hospitals)  (May-November 2016)  (iii) FGDs with 8 VHWs, 12 WDCs, 12 service users and 12 family members of the service users.  (May –November 2016) |
| Phase 2 | Testing and validation of the trust theory | i) 8 IDIs with health workers and 4 FGDs with service users (August- September 2018)  ii)Quantitative method: a household survey of 713 women  (May- June 2018) |
| Phase 3 | Theory refinement and consolidation of results into the final trust theory | Use of CMO template to visualize CMO configuration with empirical data (December 2018) |
